# Expression of the Chemokine Receptor CCR1 in Burkitt Lymphoma Cell Lines Is Linked to the CD10-Negative Cell Phenotype and Co-Expression of the EBV Latent Genes EBNA2, LMP1, and LMP2

**DOI:** 10.3390/ijms23073434

**Published:** 2022-03-22

**Authors:** Laura Zvejniece, Svetlana Kozireva, Zanna Rudevica, Ainars Leonciks, Barbro Ehlin-Henriksson, Elena Kashuba, Irina Kholodnyuk

**Affiliations:** 1 Institute of Microbiology and Virology, Riga Stradins University, 5 Ratsupites Street, 1067 Riga, Latvia; laura.zvejniece@rsu.lv (L.Z.); svetlana.kozireva@rsu.lv (S.K.); 2 Latvian Biomedical Research and Study Centre, 1 Ratsupites Street k-1, 1067 Riga, Latvia; zh_rudevica@inbox.lv (Z.R.); ainleo@biomed.lu.lv (A.L.); 3 Department of Microbiology, Tumor and Cell Biology, Biomedicum, Karolinska Institute, SE-171 65 Stockholm, Sweden; barbro.ehlin-henriksson@ki.se (B.E.-H.); elena.kashuba@ki.se (E.K.); 4 Laboratory of Molecular Mechanisms of Cell Transformation, RE Kavetsky Institute of Experimental Pathology, Oncology and Radiobiology of National Academy of Sciences of Ukraine, 45 Vasylkivska Street, UA-03022 Kyiv, Ukraine

**Keywords:** EBV, Burkitt lymphoma cell lines, CCR1, CCR2, CCR3, CCR5

## Abstract

Chemokines and their receptors regulate the migration of immune cells and the dissemination of cancer cells. CCR1, CCR2, CCR3, and CCR5 all belong to a single protein homology cluster and respond to the same inflammatory chemokines. We previously reported that CCR1 and CCR2B are induced upon Epstein-Barr virus (EBV) infection of B cells in vitro. EBV is present in almost all cases of endemic Burkitt lymphoma (BL); however, the contribution of EBV in the pathogenesis of the disease is not fully understood. Here, we analyzed the relation of the expression of *CCR1*, *CCR2*, *CCR3*, and *CCR5*, the EBV DNA load and expression of EBV latent genes in nine EBV-carrying and four EBV-negative BL cell lines. We revealed that *CCR1* is expressed at high mRNA and protein levels in two CD10-negative BL cell lines with co-expression of the EBV latent genes EBNA2, LMP1, and LMP2. Low levels of *CCR2* transcripts were found in three BL cell lines. *CCR3* and *CCR5* transcripts were hardly detectable. Our data suggest that in vivo, CCR1 may be involved in the dissemination of BL cells and in the selection of BL cells with restricted EBV gene expression programs.

## 1. Introduction

The chemokine-receptor network controls immune responses and directs the migration of immune cells. Chemokines transmit signals via chemokine receptors, which are G protein-coupled, cell-surface receptors. The C-C chemokine receptors, namely CCR1, CCR2, CCR3, and CCR5, belong to the same protein sequence homology cluster. They share responsiveness to multiple inflammatory chemokines. Inflammatory chemokines and their receptors are induced during inflammation and under many pathophysiological conditions. However, the roles of the chemokine receptors CCR1, CCR2, CCR3, and CCR5 in B lymphocytes remain unknown (reviewed in [1,2]).

In our previous work, we showed that *CCR1* and *CCR2B* (an isoform of *CCR2*) mRNA and protein expression levels are upregulated in peripheral blood (PB) B cells upon EBV infection in vitro and in established lymphoblastoid cell lines (LCLs) [3]. We also demonstrated that the CCR1 receptor was recycled on the cell surface of established LCLs in response to serum deprivation, thereby confirming that the receptor was functional. In contrast, neither the transcripts of *CCR1* or *CCR*2 nor their proteins were detected in the EBV-negative and EBV-carrying BL cell lines with latency I. Enhanced transcription of *CCR2*, *CCR7*, and *CCR9* was previously detected in tonsillar B cells upon EBV infection in vitro [4]. A recent report of time-resolved whole transcriptome sequencing of naïve B cells after EBV infection confirmed upregulation of *CCR2* starting from day 4 and after [5].

EBV, a human B lymphotropic γ-1 herpesvirus, is associated with a number of human B cell lymphoproliferative disorders (LPDs), including BL, posttransplant lymphoproliferative disease (PTLD), lymphomas in immunodeficient patients, and diffuse large B cell lymphomas (DLBCLs). In actively proliferating PTLD and LCLs generated by a viral infection of resting B cells in vitro, EBV expresses the full set of latency type III (latency III) proteins: six nuclear antigens (EBNA1, EBNA2, EBNA3A, EBNA3B, EBNA3C, and EBNA-LP) and latent membrane proteins (LMP1, LMP2A, and LMP2B) (reviewed in [6,7,8]). EBV is detected in approximately 30% of sporadic and immunodeficiency-associated BL tumors; however, EBV is found in almost all cases of endemic BL (eBL). In all BLs, constitutive activation of the *c-MYC* oncogene is a major growth-promoting event. Endemic BL classically presents as a monoclonal cell tumor, in which every cell carries the *c-MYC* translocation and EBV. Most EBV-carrying BL tumors express only one EBV protein, EBNA1, from the alternative promoter Qp (a latency I type program) [9,10,11] (reviewed in [6,7,8]). Detection of somatic mutations in immunoglobulin variable region genes [12] and gene expression arrays [13] indicated that both sporadic and eBLs were germinal center (GC)-originating B cell malignancies. Although the vast majority of eBL tumors display latency I EBV program and express the EBNA1 protein only, some studies reported that BL biopsy samples and early-passage BL cell lines are heterogeneous and express additional EBV latent and lytic cycle genes [14,15,16,17,18,19]. While some BL cell lines retain their original BL tumor cell phenotype and EBV latency I, many EBV-carrying BL cell lines shifted toward a lymphoblastoid phenotype during establishing of the culture in vitro, in the absence of the immune control, and obtained a so-called group III growth phenotype. This process was characterized by the downregulation of GC markers (including CD10 and CD77) and upregulation of the B-cell activation markers [9,11,14,20].

Up to now, not much is known about the impact of EBV on eBL disease progression. To answer the question of whether the inflammatory chemokine receptors could be implicated in the selection of the EBV-infected BL cells in vivo, we analyzed the expression pattern of the known inflammatory chemokine receptors *CCR1*, *CCR2*, *CCR3*, and *CCR5* in BL cell lines with different EBV latency programs aiming to find a putative link between the expression of particular EBV latent genes and the expression levels of the chemokine receptors.

## 2. Results

### 2.1. Characteristics of Burkitt Lymphoma Cell Lines

We analyzed the mRNA expression levels of the inflammatory chemokine receptors *CCR1*, *CCR2*, *CCR3*, and *CCR5* in 13 BL cell lines, four EBV-negative cell lines (DG75, BL41, Akata-, Mutu cl.30), and in nine EBV-carrying cell lines. The EBV-carrying BL cell lines included three type I cell lines with latency I (Rael, Akata+, Mutu cl.148), four type II cell lines (BL41/95, BL16, Jijoye P79, Akuba), and two type III cell lines (Mutu cl.99 and BL18). The Burkitt lymphoma cell lines used in the present study and references to the reported characteristics are shown in Table S1. Classification of BL cell lines as group type I, type II, or type III was based on the expression of cell surface markers, EBV-encoded proteins (EBNAs and LMPs), and growth patterns [11]. The type I BL cell lines displayed the phenotype of GC B cells, including expression of CD10; expressed the EBV latent protein EBNA1 only, and also had a single-cell suspension growth pattern. The type III BL cell lines expressed activation markers, corresponding to the immunoblastic phenotype (CD39-positive) and downregulated CD10 (the GC marker); expressed EBNA1 to EBNA5; and were growing in clumps. The type II BL cell lines maintained some GC B cell markers, including CD10, but expressed EBV genes of the latency III program [11,21,22].

**Figure 1:**
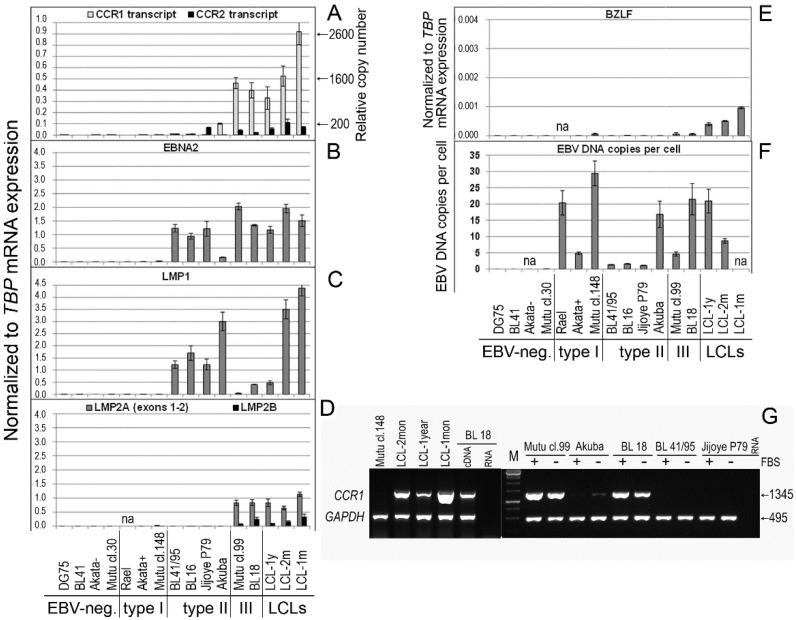
mRNA expression of the *CCR1-*, *CCR2-*, and EBV-encoded genes in Burkitt lymphoma and lymphoblastoid cell lines. The mRNA expression levels were measured using real-time RT-PCR. Shown are the mRNA expression values that are normalized to the housekeeping gene *TBP*: *CCR1* and *CCR2* (**A**); the EBV-encoded genes EBNA2 (**B**), LMP1 (**C**), LMP2A (exon 1/exon 2) and LMP2B (**D**), and BZLF (**E**). The *CCR1* transcript copy number per 1000 copies of the *TBP* transcript was assessed using quantitative real-time RT-PCR (**A**). The EBV DNA copy number per cell was quantified using the commercial EBV Real-TM Quant kit (Sacace Biotechnologies) (**F**). Shown are the EBV-negative BL cell lines: DG75, BL41, Akata-, and Mutu cl.30; the EBV-carrying BL cell lines with latency I: Rael, Akata+, and Mutu cl.148; the EBV-carrying type II BL cell lines: BL41/95, BL16, Jijoye P79, and Akuba; the EBV-carrying type III BL cell lines: Mutu cl.99 and BL18. The LCLs were established in vitro upon infection of PB B cells from different EBV-negative donors with the EBV strain B95-8: LCL-1y represents LCL that was cultured over a long period of approximately 1 year; LCL-1m is an early-passage cell line (cultured for 1–2 months); LCL-2m cells were cultured for 2–4 months. The data are presented as the mean of two-to-three independent experiments; in each experiment, samples were run in triplicate; error bars show the SD. (**G**) *CCR1* open reading frame (*CCR1*-ORF) transcripts were determined using RT-duplex PCR. First-strand cDNA templates were amplified with two pairs of primers, one pair for the housekeeping gene *GAPDH* and the other pair flanking the *CCR1*-ORF. The data are shown for the BL cell lines of the type I with EBV latency I (Mutu cl.148), type II (BL16, BL41/95, Jijoye P79), and type III (Mutu cl.99, BL18), and two LCLs (LCL-2mon, LCL-1year); FBS indicates samples after 3 h of serum deprivation (receptor recycling condition).

**Figure 2:**
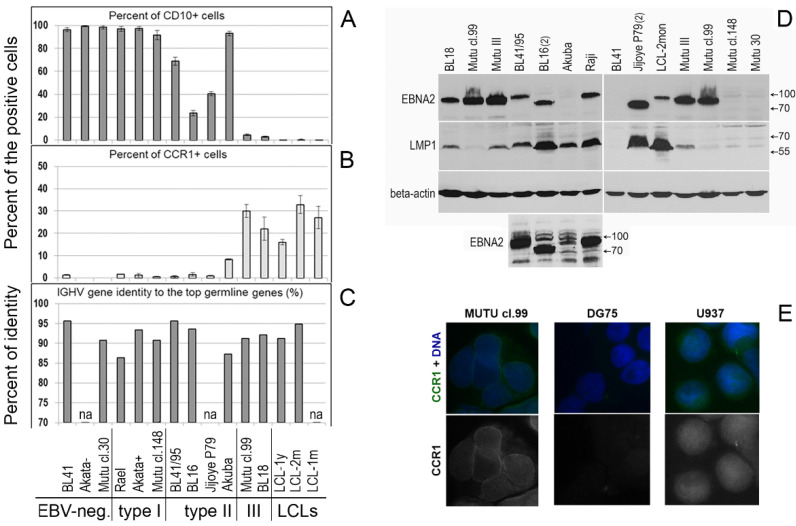
Phenotype of the Burkitt lymphoma cell lines. The percentages (%) of CD10-positive (**A**) and CCR1-expressing (**B**) cells were obtained using flow cytometry analysis. Annexin V-stained (apoptotic) and autofluorescent cells were omitted from the analysis. The presented data are from at least two independent experiments and are shown as the mean with the SD. (**C**) The IGHV gene identity (%) to the top germline genes was defined using the NCBI IGBLAST Tool. (**D**) EBNA2 and LMP1 protein expression in the type II and type III BL cell lines was assessed using immunoblotting of cell lysates and specific antibodies; the human beta-actin protein was probed as the loading control. The Raji and Mutu III BL type II cell lines served as the positive control (Table S1). All clones of the Mutu cell line are derivatives of an early-passage BL cell line [14]. BL41 is an EBV-negative cell line. A small panel, the same immunoblot with prolonged exposure, shows the EBNA2 protein in the cell lines BL41/95, BL16, Akuba, and Raji. (**E**) Immunofluorescent staining of CCR1 is shown in cells of the type III BL cell line Mutu cl.99 and human myeloid histiocytic lymphoma cell line U937, used as the positive control; in DG75, the EBV and the *CCR1* transcript negative BL cell line, the CCR1 immunofluorescent signal was not observed.

In the type II BL cell lines in our study, we did not detect LMP2A or LMP2B transcripts (Figure 1D). However, EBNA2 and LMP1 mRNA and their proteins were detected, although at varying levels (Figure 1B,C and Figure 2D). Interestingly, the EBNA2 expression level was high in Mutu cl.99 (type III), in which LMP1 expression was very low. By contrast, high LMP1 expression was observed in the type II Akuba cell line with a loss of EBNA2 expression (Figure 1B,C and Figure 2D).

### 2.2. Analyses of mRNA Expression

Low *CCR2* mRNA expression levels we detected in three BL cell lines, with the following values normalized to *TBP* mRNA expression: 0.022 ± 0.001 in BL18; 0.040 ± 0.006 in Mutu cl.99; and 0.066 ± 0.002 in Jijoye P79. By comparison, in LCL-2mon cells, the value was 0.111 ± 0.031 (Figure 1A). We also demonstrated previously that *CCR2* (isoform *CCR2B*) was expressed and that the receptor was functional in LCLs and in three endemic BL cell lines (Mutu cl.99, Mutu III, and Jijoye P79), in which the EBV-encoded latent protein EBNA2 is abundantly expressed [23] (Figure 2D).

In most BL cell lines in this study, *CCR3* mRNA expression was not detected. Traces of the *CCR3* transcripts were found in 2 BL cell lines, BL18 and Mutu cl.99, with normalized mRNA expression levels of 0.00027 ± 0.00005 and 0.0011 ± 0.0002, respectively, as well as in LCLs, with normalized mRNA expression levels of 0.00011 ± 0.00002 in LCL-1year cells and 0.00009 ± 0.00001 in LCL-2mon cells. The *CCR5* transcripts were either not found (i.e., in the EBV-negative and latency I BL cell lines) or were hardly detectable in LCLs as in the type III and type II BL cell lines. The levels of the *CCR5* normalized mRNA expression were as follows in the respective cell lines: 0.00082 ± 0.00003 in LCL-2mon; 0.00170 ± 0.00070 in LCL-1year; 0.00047 ± 0.00007 in BL18; 0.00161 ± 0.00001 in Mutu cl.99; 0.00228 ± 0.00054 in Jijoye P79; and 0.00006 ± 0.00000 in Akuba.

High levels of *CCR1* mRNA expression, with normalized expression values of 0.40 ± 0.07 and 0.46 ± 0.05, were detected in two CD10-negative type III BL cell lines, BL18 and Mutu cl.99 cells, respectively, in which the EBV latent genes EBNA2, LMP1, and LMP2A/2B were also expressed (Figure 1). In contrast to the EBV-negative BL cell lines, in which the *CCR1* transcript was not found, and the EBV type I BL cell lines, in which a few copies of the *CCR1* transcript were detected, in one CD10-positive type II BL cell line Akuba expressing EBNA2^low^ LMP1^high^ LMP2A/2B^zero^, *CCR1* mRNA expression was also detected, although at a low level, with a normalized expression of 0.10 ± 0.01 (Figure 1).

In the LCLs, the *CCR1* transcript copy number per 1000 copies of the *TBP* transcript varied from 969 ± 6 copies in the LCL-1year cells and 1650 ± 155 copies in the LCL-2mon cells to 2670 ± 398 copies in the LCL-1mon cells (Figure 1A). In the type III BL cell lines BL18 and Mutu cl.99, *CCR1* mRNA expression reached the level detected in the LCL-1year cells: the *CCR1* transcript copy number per 1000 copies of the *TBP* transcript was 1130 ± 102 copies and 1080 ± 184 copies, respectively. However, the *CCR1* mRNA expression level was 4.8- and 21.3-fold lower in the Akuba and BL41/95 type II BL lines, respectively. Notably, *CCR1* mRNA expression was abrogated in two type II BL cell lines, BL16 and Jijoye P79, which carried EBV strain Ag876 (type 2) [20]. Determination of *CCR1* mRNA expression levels and quantification of *CCR1* transcript copy number were carried out using RT real-time PCR and primers targeting exons 1 and 2. The presence of the *CCR1* ORF transcripts in the type III BL cell lines was confirmed using RT-duplex PCR (Figure 1G).

### 2.3. Analyses of Protein Expression

Immunofluorescent staining definitively detected CCR1 protein in only two BL cell lines, Mutu cl.99 and BL18 cells, which is consistent with mRNA expression data (Figure 1A and Figure 2E). The membrane signals in these BL cell lines differed from the fluorescence signals in the U937 human myeloid histiocytic lymphoma cell line, which was used as the positive control.

**Figure 3:**
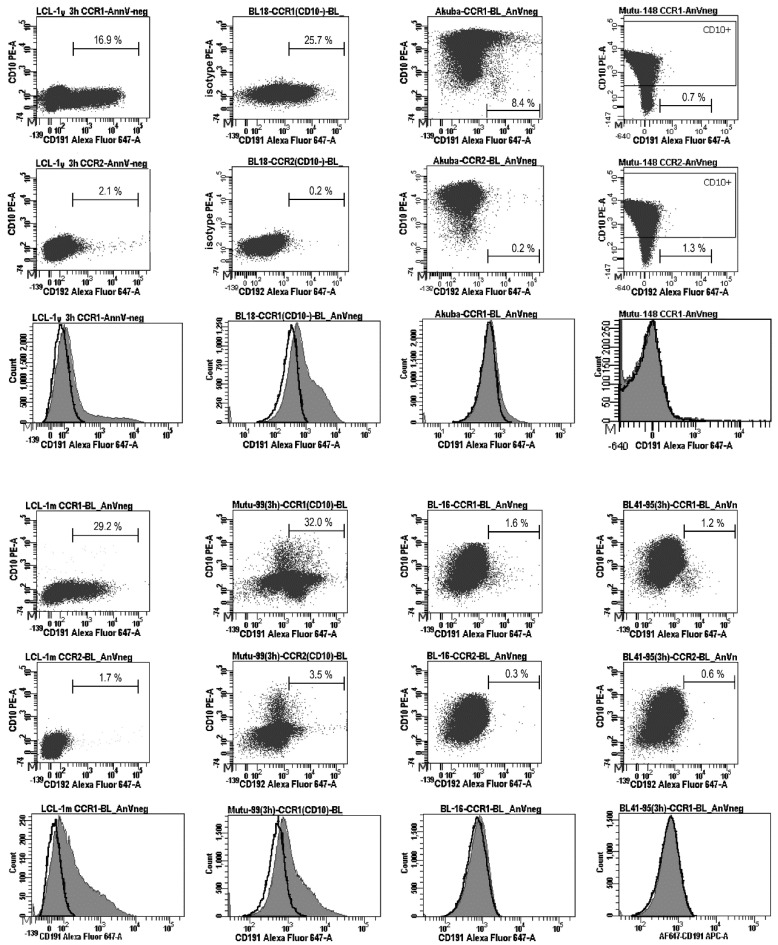
Analysis of the CCR1 cell surface expression in Burkitt lymphoma cell lines. The presence of CCR1 on the cell surface was analyzed after inducing receptor recycling (3 h of serum deprivation), using three-color flow cytometry. BL18 and Mutu cl.99 are the EBV-carrying type III BL cell lines; Akuba, BL41/95, and BL16 are the EBV-carrying type II BL cell lines; Mutu cl.148 is the EBV-carrying type I BL cell lines; LCL-1y and LCL-1m are the established in vitro LCLs, which have been cultured during approximately 1 year and 1–2 months, respectively. The dot plots show the cells stained with anti-CD10-PE, anti-CD191(CCR1)-Alexa Fluor 647, and anti-CD192(CCR2)-Alexa Fluor 647 antibodies; a minimum of 20,000 gated cells were analyzed; the threshold border was defined at 3.0% of the stained cells. Apoptotic cells (stained with annexin V-Horizon V450 or annexin V-PerCp-Cy5.5) and autofluorescent cells were excluded from the analysis. The anti-CD192(CCR2)-Alexa Fluor 647 antibodies did not detect CCR2 on the cell surface in BL cell lines nor in LCLs. The histograms show the vast majority of the CD191(CCR1)-stained cells (filled gray) shifted relative to the CD192(CCR2)-stained cells (bold lines) in LCLs and type III BL cell lines BL18 and Mutu cl.99; in the early passage type II BL cell line Akuba, only a small fraction of cells (~8.4%) are positive for CCR1. Representative results from two independent experiments are shown.

Expression of the CCR1 protein (CD191) on the cell surface and percentages (%) of CD191-positive (CD191+) and CD10-positive (CD10+) cells were determined using flow cytometry; annexin V-stained (apoptotic) cells were excluded from the analysis. The percentages of CD191+ cells were assessed after recycling of the receptor to the cell surface, as a functional response to serum deprivation, after the cells were cultured in serum-free medium for 3 h [2]. Serum deprivation resulted in the following percentages of the CCR1-positive cells: 22.0–32.0% (the average value: 27.1%), 30.0–35.7% (the average value: 32.9%), and 15.2–16.9% (the average value: 16.1%) in LCL-1m, LCL-2m, and LCL-1y, respectively, and 18.3–25.7% (the average value: 22.0%) and 28.0–32.0% (the average value: 30.0%) in the type III BL lines BL18 and Mutu cl.99, respectively (Figure 2B and Figure 3). The number of the CCR1-positive cells in the type II BL line Akuba with the CD10+ phenotype was significantly lower (the average value: 8.4%). The positivity threshold border was set at 3.0% of the stained cells according to the isotype control staining results. At the same time, the histograms demonstrate that almost all CCR1-stained cells shifted relative to the isotype- or CCR2-stained cells in LCLs and type III BL cell lines (Figure 3).

However, in Akuba cell line (the early passage type II BL cell line), only a small fraction of cells (~8.4%) carried CCR1. Cell surface expression of CCR1 was not detected in the CD10-positive and the EBV-negative (DG75, BL41, Akata-, Mutu cl.30), EBV-positive type I (Rael, Akata+, Mutu cl.148), or EBV-positive type II (BL41/95, BL16, Jijoye P79) BL cell lines (Figure 2A,B and Figure 3). We did not detect CCR2 on the cell surface of the LCL cells nor BL cells after serum deprivation. Nevertheless, using the same mouse monoclonal anti-CD192-Alexa Fluor 647 antibodies, we demonstrated the cell-surface expression of CCR2 on monocytes and B lymphocytes in PB of patients with rheumatoid arthritis [24] and patients with chronic lymphocytic leukemia [25].

### 2.4. Analyses of the EBV DNA Load and the IGHV Mutations

The EBV DNA copy number per cell (Figure 1F), expression of the immediate-early lytic EBV gene BZLF1 (Figure 1E), and the IGHV mutation status (Figure 2C) did not correlate with the expression of the EBV latent genes (EBNA2, LMP1, LMP2A, LMP2B), or with that of *CCR1* or *CCR2* (Figure 1).

## 3. Discussion

Almost all eBLs, along with the constitutively activated *c-MYC* oncogene, display the EBV latency I program expressing only one EBV latent protein EBNA1 from the Qp promoter (reviewed in [6,8]). However, the expression of other EBV latent proteins (EBNA2, LMP1, and LMP2A) was also detected in BL tumors by immunohistology [15,19] and by gene expression assays [16,17]. A proportion of eBL tumors have been identified, in which the latency III EBNA promoter Wp was active and all EBNAs, except EBNA2, were expressed (termed Wp-restricted latency). In this subset of eBLs, the EBNA2 expression was abrogated due to deletion in the EBV genome [18]. The authors concluded that eBL cells with Wp-restricted latency can be selected in vivo from progenitor cells infected with a mutated virus and suggested that the selection pressure was targeted toward downregulation of the myc proto-oncogene protein antagonist EBNA2 [18]. Subsequently, the same group isolated EBV-positive clones from an eBL that were expressing all six EBNAs in the absence of LMPs (termed EBNA2+/LMP1- latency). Notably, each form of EBV-restricted latency in the isolated cellular clones was associated with protection from apoptosis and high *c-MYC* expression [26].

The majority of BL cell lines generated from tumors with EBV latency I maintained their original cell phenotype and latency I. However, some of the BL cell lines, upon the establishing, after 20–50 passages in culture, shifted to a lymphoblastoid phenotype and expressed EBV latency III proteins (called the type III growth phenotype) [9,11,20]. For example, cell lines with the type III growth phenotype had been obtained as clones of the early-passage BL cell line Mutu [14]. 

In our previous work, we demonstrated that infection of PB B cells with EBV upregulated two inflammatory chemokine receptors, namely CCR1 and CCR2, and the expression of these chemokine receptors persisted in established LCLs [3]. In the present study, we assessed the mRNA expression levels of all known inflammatory chemokine receptors, *CCR1*, *CCR2*, *CCR3*, and *CCR5*, in 13 BL cell lines with different expression patterns of the EBV latent genes EBNA2, LMP1, LMP2A, and LMP2B in comparison with LCLs. While only trace amounts of the *CCR3* and *CCR5* transcripts were found, and low levels of *CCR2* mRNA expression were detected in type III BL cell lines and LCLs, *CCR1* mRNA expression was high in all three LCLs and in all two type III BL cell lines. Neither *CCR1* mRNA nor the CCR1 protein was detected in the EBV-negative and the latency I BL cell lines with the GC phenotype.

In this study, we quantified the *CCR1* and *CCR2* mRNA expression levels as the transcript copy number per 1000 copies of the housekeeping gene *TBP* transcript (relative copy number). While the *CCR1*-transcript relative copy number in LCLs and two type III BL cell lines ranged from 969 in LCL-1year to 2670 in LCL-1mon and from 1080 in Mutu cl.99 to 1130 in BL18, the *CCR2* mRNA expression levels, as in LCLs and in BL cell lines, were low compared to the *CCR1*. Specifically, we detected the *CCR2* relative copy numbers of only 24, 50, and 26 in LCL-1year, LCL-2mon, and LCL-1mon, respectively, and of 10 in Mutu cl.99, and 23 in Jijoye P79 (Figure 1A). Nevertheless, in our previous work [3,23], we demonstrated that the CCR2 isoform, CCR2B, was functional in LCLs and BL cell lines with co-expression of EBNA2 and LMP1 (Mutu III, Mutu cl.99, and Jijpye P79 cells), and the cells of these lines migrated toward the CCR2 unique ligand chemokine MCP1.

Here, we showed that both the *CCR1* transcript and the CCR1 protein on the cell surface were well represented in latency III LCLs and BL cell lines with the CD10-negative phenotype and concomitant co-expression of EBNA2, LMP1, and LMP2A/B. This finding is consistent with published reports demonstrating that in vitro BL cell lines that during establishing switched to latency III, display a gene expression profile consistent with a B lymphoblastoid phenotype [27], similar to those reported for LCLs generated in vitro by EBV infection of B cells [28]. Notably, *CCR1* mRNA and CCR1 protein cell-surface expression were also detected, although at a very low level, in the type II BL cell line with the GC-like CD10-positive cell phenotype—in the early-passage Akuba cell line expressing EBNA2^low^ LMP1^high^ LMP2A/2B^zero^. This finding is consistent with previous observations and the notion reported by Kelly and coauthors [18,26] that in vivo selection of eBL cells is directed against the simultaneous expression of EBNA2 and LMP1. In Akuba, only a small fraction of cells (~8.4%) presented the CCR1 protein on the cell surface demonstrating the heterogeneity of this early-passage BL cell line (Figure 3).

The *CCR1*, *CCR2*, *CCR3*, and *CCR5* genes reside in the same region of human chromosome 3 3p21.31 [29,30]. There is high DNA and protein sequence identity between these four inflammatory chemokine receptors, namely CCR1, CCR2, CCR3, and CCR5, and these receptors respond to the same inflammatory chemokine ligands [2]. The CCR1, CCR2, CCR3, and CCR5 chemokine ligands CCL3/MIP-1α (macrophage inflammatory protein-1α), CCL5/RANTES (regulated upon activation normal T cell expressed and secreted), and CCL8/MCP-2 (monocyte chemotactic protein-2) are expressed and secreted by inflammatory macrophages, CD14+/CD16- classical monocytes, cytotoxic CD56-dim NK cells, mature neutrophils, and CD4+ and CD8+ alpha-beta T cells at high and medium levels (reviewed in [1,2,31] and, thus, attract these immune cells to receptor-presenting targets. In immunocompetent patients, clearing CCR1-presenting BL cells by immune cells may be one of the mechanisms underlying in vivo selection of BL cells with EBV-restricted latencies (EBNA1 only, EBNA2-deleted, EBNA2+/LMP1-deleted). Furthermore, the CCR1 chemokine ligands (endogenous agonists) are abundantly secreted in bone marrow (CCL3, CCL7), lymph nodes (CCL4, CCL14), spleen (CCL3, CCL8, CCL14), tonsil (CCL8), and liver (CCL14, CCL15, CCL16) (reviewed in [1,2,31,32]). Consequently, in immunocompromised patients or during transient immunodeficiency, chemotactic migration of the CCR1-expressing BL cells into secondary organs could impact disease pathogenesis.

Concluding, we demonstrated that only one CCR1 out of four known chemokine receptors in the family of inflammatory chemokine receptors was highly upregulated in the EBV-carrying LCLs and BL cell lines with co-expression of the EBV latent genes EBNA1, EBNA2, LMP1, and LMP2A/2B. In the CD10-positive BL cell lines, EBV-negative and EBV-positive with latency I program, none of the inflammatory chemokine receptors was expressed, which suggests that in vivo CD10-positive BL cells escape chemokine ligand-expressing immune cells, such as macrophages, T- and NK-lymphocytes. Our results may help to reveal mechanisms of endemic BL lymphomagenesis, contributing to understanding the selection of EBV-restricted latency BL-cell subsets within a tumor mass. We also speculate that in vivo endemic BL cells expressing CCR1 are prone to migrate toward ligand-expressing immune cells and ligand-rich blood organs, and therefore, in an immunocompromised state, dissemination of malignant cells may contribute to the pathogenesis of this cancer.

## 4. Materials and Methods

The BL cell lines analyzed in this study along with the references to their origin, expression of EBV latent genes, and their reported characteristics are listed in Table S1. The BL cell lines were obtained from the collection at the Department of Microbiology, Tumor and Cell Biology of the Karolinska Institute (Stockholm, Sweden) and included four EBV-negative and nine EBV-carrying BL cell lines: three type I, four type II, and two type III lines (Figure 1). The BL cell line group types (I, II, and III) were defined according to the phenotype characteristics and expression of EBV latent genes [11,22]. Type I cells have a GC phenotype and EBV latency I; type III cells display a lymphoblastoid phenotype and express EBV latent genes; and type II cells represent an intermediate phenotype expressing the GC marker CD10 and EBV latent genes. All BL cell lines in this report, except for Akuba, carry the t(8;14) translocation [21,33] and have been studied over prolonged periods. The Akuba cell line carries the t(8;22) translocation [21,33]. We used an early-passage Akuba cell line that was recovered from stock frozen in 1972. All EBV-positive cell lines expressed EBNA1 (Table S1).

The mRNA expression levels of the *CCR1*, *CCR2*, *CCR3*, and *CCR5* genes and the EBV genes, EBNA2, LMP1, LMP2A, LMP2B, and BZLF1, were assessed using real-time RT-PCR with the PerfeCTa SYBR Green FastMix (Quanta BioSciences Inc., Beverly, MA, USA) and the CFX96 Touch Real-Time PCR detection system (Bio-Rad Laboratories Inc., Richmond, CA, USA). The mRNA expression levels of the genes of interest were normalized to the mRNA expression levels of the housekeeping gene *TBP* (encoding TATA box-binding protein). The presence of the *CCR1* open reading frame (ORF) transcript was determined using RT-duplex PCR performed with two pairs of primers flanking the *CCR1* ORF and matching the housekeeping gene *GAPDH* (glyceraldehyde-3-phosphate dehydrogenase). We previously described the real-time RT-PCR procedure, the RT-duplex PCR protocol, and the corresponding primers in prior publications [3,30]. The EBV-encoded EBNA2, LMP1, and BZLF1 (exon 2/exon 3) transcripts were assessed using previously reported primers [34]; the EBV type 2 EBNA2 (in Jijoye P79 and BL16) and LMP2A (exon 1/exon 2 and exon 1/exon 3) transcripts were measured using primers described by Bell and coauthors [35]; and the LMP2B primers were described in a report by Fox and coauthors [36].

The copy numbers of the *CCR1* and *TBP* transcripts were determined by quantitative real-time RT-PCR. For copy number quantification, we applied standard curves based on serial dilutions of the plasmids with the inserted *CCR1* or *TBP* transcripts derived from the established LCL-2mon reference line. The transcripts were cloned into the pCR-TOPO vector (TOPO TA Cloning Kit, Invitrogen, Thermo Fisher Scientific Inc., Waltham, MA, USA) and sequenced for verification. The EBV DNA copy number per cell for each cell line was quantified using the EBV Real-TM Quant kit (Sacace Biotechnologies, Como, Italy).

The analysis of the immunoglobulin heavy-chain variable region (IGHV) gene mutations was carried out according to the described protocol [37] using six 5′ IGHV leader primers and four 3′ IGHJ primers. The percentage (%) of the identity shared with the top germline genes was determined by applying the NCBI IGBLAST Tool (https://www.ncbi.nlm.nih.gov/igblast/, accessed on 1 January 2021).

Western blot analyses were performed, using whole-cell lysates, with a mouse monoclonal anti-EBNA2 antibody (clone PE2, ab90543, Abcam, Cambridge, UK) and a mouse monoclonal anti-LMP1 antibody (clone CS 1-4, ab78113, Abcam, Cambridge, UK). CCR1 immunostaining was performed using goat polyclonal anti-human CCR1 antibodies (CKR-1, sc-6125, Santa Cruz Biotechnology, Inc., Dallas, TX, USA) and donkey anti-goat IgG, F(ab’)- fragment antibody conjugated with FITC (sc-3853, Santa Cruz Biotechnology, Inc., Dallas, TX, USA). DNA in the cells, fixed with a mixture of methanol and acetone (1:1), was stained using Hoechst 33258 (Sigma-Aldrich Co. LLC., Saint Louis, MO, USA).

For the three-color flow cytometry analysis, cells were stained with a mouse monoclonal anti-CD191 antibody (anti-CD191-Alexa Fluor 647, clone 53504) targeting the cell surface CCR1 or with a mouse monoclonal anti-CD192 antibody (anti-CD192-Alexa Fluor 647, clone 48607) targeting the cell surface CCR2 and a mouse monoclonal anti-CD10 (anti-CD10-PE, clone HI10a), according to a standard protocol [3]; anti-mouse IgG2b-Alexa Fluor 647 was used for the isotype control. Apoptotic cells were stained with annexin V-Horizon V450 or annexin V-PerCp-Cy5.5 (BD Biosciences Pharmingen, Palo Alto, CA, USA). Autofluorescent and apoptotic cells were excluded from the analyses. The stained cells were analyzed using a BD FACSAria III flow cytometer (Becton Dickinson and Company, Franklin Lakes, NJ, USA). In the CCR1 cell-surface expression analysis, cell lines were assessed the day after supplementation with fresh medium, during the late exponential growth phase. To induce receptor recycling to the cell surface, the cells were cultivated in serum-free medium for 3 h, and then, the number of receptor-expressing cells was counted using flow cytometry.

## Data Availability

The data that support the findings of this study are available upon request.

## Supplementary Materials

The following supporting information can be downloaded at: https://www.mdpi.com/article/10.3390/ijms23073434/s1.
