# Supplementary material for: Expression of the Chemokine Receptor CCR1 in Burkitt Lymphoma Cell Lines Is Linked to the CD10-Negative Cell Phenotype and Co-Expression of the EBV Latent Genes EBNA2, LMP1, and LMP2
Source: Int J Mol Sci. 2022 Mar 22;23(7):3434. doi: 10.3390/ijms23073434 (PMC8998437; doi:10.3390/ijms23073434)
Supplement: Supplementary file 1 [file ijms-23-03434-s001.zip › ijms-1618074-supplementary.pdf]

Table S1. Burkitt lymphoma cell lines analyzed in the present study

Name	Origin: Age / sex / ethnic	Origin: sample / cell line	EBV status / phenotype group[a]	EBV latency / EBNA proteins[b]	Reference: author of the original characteristics	Reference: reported characteristics
DG-75	10 / XY / Jewish; Ashkenazi (sporadic)	pleural effusion	Negative	–	[1] Ben-Bassat *et al,* 1977	[12] Pokrovskaja *et al,* 1996;<br>[13] Nagy *et al.,* 2000;<br>[14] Maria Murga Penas *et al,* 2014;<br>[15] Mushtaq et al, 2015
BL41	8 / XY / Caucasian (sporadic)	tumor	Negative	–	[2] Lenoir *et al,* 1982	[12] Pokrovskaja *et al,* 1996;<br>[13] Nagy *et al.,* 2000;<br>[14] Maria Murga Penas *et al,* 2014;<br>[15] Mushtaq et al, 2015;<br>[16] Ehlin-Henriksson *et al,* 1987;
BL41/95	8 / XY / Caucasian (sporadic)	tumor	Positive / converted with EBV / type II	EBNA1, EBNA2, LMP1	[3] Lenoir *et al,* 1985	[12] Pokrovskaja *et al,* 1996;<br>[15] Mushtaq et al, 2015;<br>[16] Ehlin-Henriksson *et al,* 1987;<br>[17] **Torsteinsdottir *et al,* 1989; Present study**
Akata-	4 / XX / Japanese	Akata+ cell line	Negative	–	[4] Shimizu *et al,* 1994	[15] Mushtaq et al, 2015;<br>[18] Nagy *et al.,* 2002
Mutu cl.30	n.a. / XY / African (endemic)	Mutu I cell line	Negative	–	[5] Chodosh *et al,* 1998	[15] Mushtaq et al, 2015;<br>[18] Nagy *et al.,* 2002
Rael	n.a. / XY / African (endemic)	tumor	Positive / type I	Latency I / EBNA1 only	[6] Klein *et al,* 1972	[12] Pokrovskaja *et al,* 1996;<br>[14] Maria Murga Penas *et al,* 2014;<br>[15] Mushtaq et al, 2015;<br>[16] Ehlin-Henriksson *et al,* 1987;<br>[18] Nagy *et al.,* 2002<br>[19] Minarovits *et al,* 1991;<br>[20] **Altiok *et al,* 1992**
Akata+	4 / XX / Japanese	tumor	Positive / type I	Latency I / EBNA1 only	[7] Takada *et al,* 1991	[13,18] Nagy *et al.,* 2000; 2002;<br>[15] Mushtaq et al, 2015;<br>[20] **Altiok *et al,* 1992;**<br>[21] Karpova et al., 2006
Mutu cl.148	n.a. / XY / African (endemic)	tumor / early passage cell line	Positive / type I	Latency I / EBNA1 only	[8] **Gregory *et al,* 1990**	[12] Pokrovskaja *et al,* 1996;<br>[13] Nagy *et al.,* 2000;<br>[15] Mushtaq et al, 2015;<br>[20] **Altiok *et al,* 1992**
BL16	5 / XX /	tumor	EBV type 2	EBNA1,	[3] Lenoir *et al,* 1985;	[12] Pokrovskaja *et al,* 1996;

	African, Reunion Island (sporadic)		positive / type II	EBNA2, LMP1	[9] Bernheim et al.,1983	[14] Maria Murga Penas *et al,* 2014;<br>[15] Mushtaq et al, 2015;<br>[16] Ehlin-Henriksson *et al,* 1987;<br>[22,23] **Rowe *et al,* 1987; 1989;**<br>[24] Falk *et al,* 1997;<br>**Present study**
Jijoye P79	7 / XY / African (endemic)	ascites / Jijoye cell line	EBV type 2 positive / type II	EBNA1, EBNA2, EBNA3abc, LMP1	[10] Pulvertaft *et al,* 1965	[15] Mushtaq et al, 2015;<br>[18] Nagy *et al.,* 2002;<br>[19] Minarovits *et al,* 1991;<br>[20] **Altiok *et al,* 1992;**<br>[23] Rowe *et al,* 1989;<br>[24] Falk *et al,* 1997;<br>**Present study**
Akuba	n.a. / XX / African (endemic)	tumor	Positive / type II	EBNA1, EBNA2^{low} LMP1	[6] Klein *et al,* 1972;<br>[11] **Ernberg and Moar, 1981**	[14] Maria Murga Penas *et al,* 2014;<br>[16] Ehlin-Henriksson *et al,* 1987<br>**Present study**
Mutu cl.99	n.a. / XY / African (endemic)	tumor / early passage cell line	Positive / type III	EBNA1, EBNA2, EBNA3abc, EBNA-LP LMP1^{low} , LMP2A	[8] **Gregory *et al,* 1990**	[12] Pokrovskaja *et al,* 1996;<br>[15] Mushtaq et al, 2015;<br>[20] **Altiok *et al,* 1992;**<br>**Present study**
BL18	3 / XY / North-African (sporadic)	tumor	Positive / type III	EBNA1, EBNA2, EBNA3, LMP1, LMP2A	[3] Lenoir *et al,* 1985;<br>[9] Bernheim et al.,1983	[14] Maria Murga Penas *et al,* 2014;<br>[15] Mushtaq et al, 2015;<br>[16] Ehlin-Henriksson *et al,* 1987;<br>[22] **Rowe *et al,* 1987;**<br>**Present study**

[a] Definition of the type I and type III groups was based on the phenotype characteristics; the type I BL cells displayed the phenotype of germinal center (GC) B cells, whereas the type III cells expressed activation markers corresponding to the lymphoblastoid phenotype [16,22]. [b] The data on the expression of EBNAs are taken from the references in **Bold**; n.a., not available.

The Mutu clone (cl.) cell lines were derived from an early passage BL cell line: Mutu cl.148 with the type I phenotype and the type III Mutu cl.99 were obtained by selection and *in vitro* culture [8]. Mutu III is a derivative of the type I Mutu clone cell line that had converted *in vitro* to the phenotype group type II and expressed EBNA2 and LMP1 [21,25]. Mutu cl.30 is an EBV-negative BL line obtained by hydroxyurea treatment of parental Mutu I cells. Akata- is a variant of the original BL Akata cell line with latency I that spontaneously lost EBV in culture. The BL41/95 cell line was generated by infection of the EBV-negative BL41 cells with the B95-8 (type 1) EBV strain. Jijoye P79 and BL16 carry the EBV strain type 2 [23]. All BL cell lines in the study, except Akuba, carry the t(8;14) translocation [14,16] and have been studied for prolonged periods. The Akuba cell line carries the t(8;22) translocation [14,16]. In the present study, the early-passage Akuba cell line, recovered from an original frozen stock in 1972, was used.

References

1. Ben-Bassat, H.; Goldblum, N.; Mitrani, S.; Goldblum, T.; Yoffey, J.M.; Cohen, M.M.; Bentwich, Z.; Ramot, B.; Klein, E.; Klein, G. Establishment in continuous culture of a new type of lymphocyte from a "Burkitt like" malignant lymphoma (line D.G.-75). *Int J Cancer* **1977**, *19*, 27–33. doi: 10.1002/ijc.2910190105.
2. Lenoir, G.M.; Preud'homme, J.L.; Bernheim, A.; Berger, R. Correlation between immunoglobulin light chain expresion and variant translocation in Burkitt's lymphoma. *Nature* **1982**, *298*, 474–476. doi: 10.1038/298474a0.
3. Lenoir, GM.; Vuillaume, M.; and Bonnardel, C. Burkitt's lymphoma: a human cancer model. *IARC Sci. Publ.* **1985,** *60*, 309-18. PMID: 3934070
4. Shimizu, N.; Tanabe-Tochikura, A.; Kuroiwa, Y.; Takada, K. Isolation of Epstein-Barr virus (EBV)-negative cell clones from the EBV-positive Burkitt lymphoma (BL) line Akata: malignant phenotypes of BL cells are dependent on EBV. *J. Virol.* **1994**, *68*(9), 6069 –73. doi: 10.1128/JVI.68.9.6069-6073.1994
5. Chodosh, J.; Holder, VP.; Gan, YJ.; Belgaumi, A.; Sample, J.; Sixbey, JW. Eradication of latent Epstein-Barr virus by hydroxyurea alters the growth-transformed cell phenotype. *J. Infect. Dis.* **1998**, *177*, 1194 –201. doi: 10.1086/515290.
6. Klein, G.; Dombos, L and Gothoskar, B. Sensitivity of Epstein-Barr virus (EBV) producer and non-producer human lymphoblastoid cell lines to superinfection with EB-virus. *Int. J. Cancer* **1972**, *10*, 44-57. doi: 10.1002/ijc.2910100108
7. Takada, K.; Horinouchi, K.; Ono, Y.; Aya, T.; Osato, T.; Takahashi, M.; Hayasaka, S. An Epstein-Barr virus-producer line Akata: establishment of the cell line and analysis of viral DNA. *Virus Genes* **1991**, *5*, 147–56. doi: 10.1007/BF00571929.
8. Gregory, C.D.; Rowe, M.; Rickinson, A.B. Different Epstein-Barr virus-B cell interactions in phenotypically distinct clones of a Burkitt's lymphoma cell line. *J. Gen. Virol.* **1990**, *71*, 1481–1495. doi: 10.1099/0022-1317-71-7-1481.
9. Bernheim, A.; Berger, R.; Lenoir, G. Cytogenetic studies on Burkitt's lymphoma cell lines. Cancer Genet. Cytogenet. **1983**, *8*(3), 223-9. doi: 10.1016/0165-4608(83)90138-3.
10. Pulvertaft, R.J.V. A study of malignant tumors in Nigeria by short term tissue culture. *J. Clin. Path.* **1965**, *18*, 261–73. doi: 10.1136/jcp.18.3.261.
11. Ernberg, I.; Moar, M.H. Induction of Epstein-Barr virus early antigen, virus capsid antigen and virus DNA synthesis by mitomycin C. *J. Gen. Virol.* **1981**, *52*(Pt 1), 39-48. doi: 10.1099/0022-1317-52-1-39.
12. Pokrovskaja, K.; Ehlin-Henriksson, B.; Bartkova, J.; Bartek, J.; Scuderi, R.; Szekely, L.; Wiman, K.G.; Klein, G. Phenotype-related differences in the expression of D type cyclins in human B cell-derived lines. *Cell Growth Differ.* **1996**, *7*, 1723–1732. PMID: 8959341.
13. Nagy, N.; Cerboni, C.; Mattsson, K.; Maeda, A.; Gogolák, P.; Sümegi, J.; Lányi, A.; Székely, L.; Carbone, E.; Klein, G.; Klein, E. SH2D1A and SLAM protein expression in human lymphocytes and derived cell lines. *Int. J. Cancer* **2000**, *88*(3), 439-47. PMID: 11054674
14. Penas, M.E.M.; Schilling, G.; Behrmann, P.; Klokow, M.; Vettorazzi, E.; Bokemeyer, C.; Dierlamm, J. Comprehensive cytogenetic and molecular cytogenetic analysis of 44 Burkitt lymphoma cell lines: Secondary chromosomal changes characterization, karyotypic evolution, and comparison with primary samples. *Genes Chromosomes Cancer* **2014**, *53*, 497–515. doi: 10.1002/gcc.22161.
15. Mushtaq, M.; Darekar, S.; Klein, G.; Kashuba, E. Different mechanisms of regulation of the warburg effect in lymphoblastoid and Burkitt lymphoma cells. *PLos One* **2015**, *10*(8), e0136142. doi: 10.1371/journal.pone.0136142.
16. Ehlin-Henriksson, B.; Manneborg-Sandlund, A.; Klein, G. Expression of B-cell-specific markers in different Burkitt lymphoma subgroups. *Int. J. Cancer* **1987**, *39*, 211–218. doi: 10.1002/ijc.2910390215.
17. Torsteinsdottir, S.; Andersson, M.; Avila-Carino, J.; Ehlin-Henriksson, B.; Masucci, M. G.; Klein, G. and Klein, E. Reversion of tumorigenicity and decreased agarose clonability after EBV conversion of an IgH/myc translocation carrying BL line. *lnt. I. Cancer* **1989**, *43*(2), 273-8. doi: 10.1002/ijc.2910430219.
18. Nagy N.; Maeda A.; Bandobashi K.; Kis LL.; Nishikawa J.; Trivedi P.; Faggioni A.; Klein G.; Klein E. SH2D1A expression in Burkitt lymphoma cells is restricted to EBV positive group I lines and is downregulated in parallel with immunoblastic transformation. *Int. J. Cancer* **2002**, *100*(4), 433-40. doi: 10.1002/ijc.10498.
19. Minarovits, J.; Minarovits-Kormuta, S.; Ehlin-Henriksson, B.; Falk, K.; Klein, G.; Ernberg, I. Host cell phenotype-dependent methylation patterns of Epstein-Barr virus DNA. *J. Gen. Virol.* **1991**, *2* (Pt 7), 1591-9. doi: 10.1099/0022-1317-72-7-1591.
20. Altiok, E.; Minarovits, J.; Hu, LF.; Contreras-Brodin, B.; Klein, G.; Ernberg, I. Host-cell-phenotype-dependent control of the BCR2/BWR1 promoter complex regulates the expression of Epstein-Barr virus nuclear antigens 2-6. *Proc. Natl. Acad. Sci. U S A.* **1992**, *89*(3), 905-9. doi: 10.1073/pnas.89.3.905.

21. Karpova, M.B.; Schoumans, J.; Blennow, E.; Ernberg, I.; Henter, JI.; Smirnov, AF.; Nordenskjöld, M.; Fadeel, B. Combined spectral karyotyping, comparative genomic hybridization, and in vitro apoptyping of a panel of Burkitt's lymphoma-derived B cell lines reveals an unexpected complexity of chromosomal aberrations and a recurrence of specific abnormalities in chemoresistant cell lines. *Int J Oncol*. **2006**, *28*(3), 605-17. PMID: 16465364
22. Rowe, M.; Rowe, D.T.; Gregory, C.D.; Young, L.S.; Farrell, P.J.; Rupani, H.; Rickinson, A.B. Differences in B cell growth phenotype reflect novel patterns of Epstein-Barr virus latent gene expression in Burkitt's lymphoma cells. *EMBO J.* **1987**, *6*, 2743–2751.
23. Rowe, M.; Young, L.S.; Cadwallader, K.; Petti, L.; Kieff, E.; Rickinson, A.B. Distinction between Epstein-Barr virus type A (EBNA 2A) and type B (EBNA 2B) isolates extends to the EBNA 3 family of nuclear proteins. *J. Virol.* **1989**, *63*, 1031–1039. doi: 10.1128/JVI.63.3.1031-1039.1989.
24. Falk, K.I.; Zou, J.Z.; Lucht, E.; Linde, A.; Ernberg, I. Direct identification by PCR of EBV types and variants in clinical samples. *J Med Virol* **1997**, *51*(4), 355-63. doi: 10.1002/(sici)1096-9071(199704)51:4<355::aid-jmv15>3.0.co;2-h.
25. Szeles, A.; Falk, KI.; Imreh, S. and Klein, G. Visualization of alternative Epstein-Barr virus expression programs by fluorescent *in situ* hybridization at the cell level. *J. Virol.* **1999**, *73*, 5064-5069. doi: 10.1128/JVI.73.6.5064-5069.1999.
